# The Study of Cyclosporin A Nanocrystals Uptake and Transport across an Intestinal Epithelial Cell Model

**DOI:** 10.3390/polym14101975

**Published:** 2022-05-12

**Authors:** Wenjun Sun, Yang Tian, Zengming Wang, Hui Zhang, Aiping Zheng

**Affiliations:** Institute of Pharmacology and Toxicology, Academy of Military Medical Sciences, Beijing 100850, China; swj2008113104@163.com (W.S.); tianyang1127@126.com (Y.T.); wangzm.1986@163.com (Z.W.)

**Keywords:** cyclosporin A nanocrystals, endocytosis inhibitor, cellular uptake, Caco-2 cell monolayer

## Abstract

Cyclosporin A nanocrystals (CsA-NCs) interaction with Caco-2 cells were investigated in this study, including cellular uptake and transport across Caco-2 cell monolayers. CsA-NCs of 165 nm, 240 nm and 450 nm were formulated. The dissolution of CsA-NCs was investigated by paddle method. The effect of size, concentration and incubation time on cellular uptake and dissolution kinetics of CsA-NCs in cells were studied. Uptake mechanisms were also evaluated using endocytotic inhibitors and low temperature (4 °C). The cell monolayers were incubated with each diameter CsA-NCs to evaluate the effect of size on the permeation characteristics of CsA across the intestinal mucosa. The results of dissolution study showed that 165 nm CsA-NC had the highest dissolution rate followed by 240 CsA-NC and finally 450 nm CsA-NC. The saturation of cell uptake of CsA-NCs was observed with the increase of incubation concentration and time. 240 nm and 450 nm CsA-NCs had the lowest and highest uptake efficiency at different time and drug concentration, respectively. The uptake of all three-sized CsA-NCs declined significantly in some different degree after the pre-treatment with different endocytosis inhibitors. 165 nm CsA-NC showed a highest transport capacity across monolayers at the same concentration and time. The results suggest that the size of CsA-NCs can not only affect the efficiency of cellular uptake, but also the type of endocytosis. Decreasing particle size of CsA-NCs can improve transport capacity of CsA through cell monolayer.

## 1. Introduction

Many compounds with good pharmacological activity are difficult to been developed into new drugs due to their poor solubility. Hence, many strategies were proposed to improve the solubility and dissolution of insoluble drugs, such as salt formation, solubilization, cyclodextrin, solid dispersion, liposomes, micelles, solid lipid nanoparticles, and so on. However, these approaches have some problems, such as solvent toxicity, low drug load, poor physical and chemical stability, and difficulty in industrial production. Drug nanocrystals are typically composed of drug particles with an average diameter of less than 1 µm and tiny amounts of stabilizers (surfactants or polymers). An increase in the surface area improves the dissolution rate and equilibrium solubility of drug nanocrystals, as described by the Noyes-Whitney equation and Ostwald-Freundlich equation [1,2]. Compared with the above methods, nanocrystals have a high drug load with a good safety. In addition, the preparation of nanocrystals is easy to industrialize. Therefore, drug nanocrystals have attracted extensive attention in the delivery of insoluble drugs.

Intact nanocrystals of insoluble drug can survive in the body for a long time. A long biological life of intact hybrid nanocrystals of quercetin and cyclosporin A was observed in the gastrointestinal tract of Sprague-Dawley (SD) rats by live imaging [3,4]. Transmission electron microscopy (TEM) showed that intact hybrid paclitaxel could be directly absorbed by epithelium [5]. Confocal images of nasopharyngeal epidermal carcinoma (KB) cells cultured with THPE nanocrystals showed the observed fluorescence within cells, which verified that the intact THPE nanocrystals could transfer across KB cell membranes [6]. Li et al. [7] also utilized the hybrid nanocrystal concept demonstrated that the intact drug nanocrystals were taken up directly by the cells, and subsequently dissolved in the cytoplasm. Eukaryotic cells use a variety of pathways to internalize substances, including phagocytosis, macrophytic endocytosis, clathrin-mediated endocytosis, and non-clathrin-mediated endocytosis. It is thought that cells use these different mechanisms to accomplish different tasks [8]. The mediated pathways of endocytosis of nanoparticle can be analyzed by adding inhibitors of different endocytosis pathways [9]. It’s reported that the physical properties, such as particle size, surface charge, hydrophobicity and so on are the factors which affect the gastrointestinal (GI) uptake of nanoparticles [10]. Therefore, it is important to investigate the factors influencing the GI uptake of nanoparticles in order to obtain an efficient nanoparticle based oral drug delivery system including for oral drug nanocrystals.

The small intestine is a main organ in which the nutrients and drugs were absorbed and transported into circulatory system. The small intestine has extremely complexed environment, including chemical reactions, enzymes actions, and probiotic activities [11]. Thus, the suitable model in vitro should be established to mimic normal intestinal epithelium. In 2009, Sato et al., cultured the first organoids from outbred mice intestinal stem cells [12]. The organoids contain enterocytes, goblet cells, Paneth cells, and enteroendocrine cells surrounding the lumen to mimic the regular intestinal epithelium structure [13]. In many studies [11,13,14,15], The organoid has been utilized as a model to mimic the functions of the intestinal epithelium to evaluate the properties of nanoparticle-based drug delivery. Caco-2 cells originate from the human colon adenocarcinoma cell line. It can express transporter proteins, efflux proteins (P-glycolprotein) and cytochrome P450 isozymes [16]. After about three weeks of culture, the Caco-2 cells differentiate to form enterocyte-like cell monolayer with tight junction, micro villi and brush border [17]. The Caco-2 cell monolayer can mimics the intestinal epithelium and has been used extensively for the high-throughput screening of drug permeability prior to in vivo studies. Considering the origin, Caco-2 cells can eliminate the differences between species [18], and make the results of in vitro studies have a good correlation and reproducibility with human studies [19].

CsA is a non-polar cyclic undecapeptide with unique immunosuppressive properties. It has been extensively used to treat organ transplantation and autoimmune diseases [20]. The molecular weight of CsA is 1202.61. CsA possesses low water solubility (27.67 μg/mL at 25 °C) and high lipophilicity (log P = 2.92 at pH 7.4) [21]. Due to the rigid cyclic structure of CsA and its high molecular weight, CsA also exhibits very low permeability across almost all the biological barriers, such as the gastrointestinal tract, skin, and cornea [22].

In this present study, we have used Caco-2 cell as an in vitro model to investigate the uptake of CsA-NCs. CsA-NCs were prepared by the wet bead milling method with particle sizes of ~165 nm, ~240 nm, and ~450 nm. Cellular uptake, uptake mechanism and transport across Caco-2 cell monolayer experiments were conducted to gain insight into the size effect of CsA-NCs on their interaction with Caco-2 cells.

## 2. Materials and Methods

### 2.1. Materials

CsA was purchased from Taishang Chemical Pharmacy Company (Taishang, China). Cyclosporin D (CsD) was purchased from Shanghai Tongtian Biotechnology Company (Shanghai, China). Vitamin E polyethylene glycol succinate (TPGS) was purchased from Shanghai Aladdin Biochemical Technology Co., Ltd. (Shanghai, China). Sodium dodecyl sulfate (SDS) was purchased from BASF, Ludwigshafen, Germany. Hydroxypropyl cellulose (HPC) was purchased from Nippon Soda Co., Ltd. (Tokyo, Japan). Roswell Park Memorial Institute (RPMI) 1640 medium, phosphate buffered saline (PBS) and Hank’s Balanced Salt Solution (HBSS) were purchased from HyClone Co., Ltd. (Logan, UT, USA). Fetal bovine serum (FBS) was purchased from gibco Co., Ltd. (GrandIsland, NY, USA). PMSF and RIPA buffer were purchased from Thermo Fisher Scientific Inc. (Rochester, NY, USA). The purified water used in this study was prepared using a Mille-Q system (EMD Millipore, Billerica, MA, USA). HPLC-grade acetonitrile and methanol were obtained from Thermo Fisher Scientific (Waltham, MA, USA). All other reagents were analytical grade.

### 2.2. Preparation of the CsA-NCs

CsA-NCs with different particle sizes were prepared by the wet bead milling method. HPC (7.5 g), TPGS (1.0 g) and SDS (0.1 g) were dissolved in deionized water (100 mL) to form a surfactant solution. CsA (10 g) was added to the surfactant solution and stirred at 300 rpm with a magnetic agitator. The uniform CsA suspensions with the surfactants were prepared by a high-shear apparatus (Ultra Turrax, T25, IKA, Staufen, Germany) at 10,000 rpm for 5 min. The uniform suspensions were then milled using the grinding machine (Dyno^®^-Mill Research Lab, WAB, Basel Region, Switzerland) at 1500, 2000, and 2500 rpm for 5 min, followed by 3000 rpm for different periods (0.5 h, 1.5 h and 4.0 h) to prepare CsA-NCs with the different particle sizes (160 nm, 240 nm and 450 nm). The 0.1 mm yttrium-stabilized zirconium beads were used as milling media in this study.

### 2.3. Particle Characterization

The mean particle size and polydispersity index (PDI) of CsA-NCs were detected by photon correlation spectroscopy (PCS), using a Malvern Zetasizer (ZS-90; Malvern Instruments, Malvern, UK). Samples were diluted in water to a suitable concentration. The optical parameters of cyclosporin A were: real refractive index (RI) 1.49 and imaginary refractive index (IRI) 0.03 [23].

### 2.4. Characterization of CsA NCs

The morphologies of CsA -NCs (165 nm, 240 nm and 450 nm) were determined by scanning electron microscopy (SEM) (JSM-7900F, JEOL, Tokyo, Japan). Samples were affixed to aluminum stubs using a double-sided carbon tape and sputter-coated with gold under an argon atmosphere. Differential scanning calorimetry (DSC) was performed with a DSC 214 differential scanning calorimeter (NETZSCH, Bavaria, Germany). The thermal properties of CsA, the stabilizers (HPC, TPGS and SDS), the physical mixture (raw CsA and stabilizers), and the CsA-NCs with different particle sizes were analyzed. Accurately weighted samples of 3 mg were placed in open aluminium pans, and DSC scans were recorded at a heating rate of 10 K/min from 20 °C to 200 °C under nitrogen purge gas flow (20 mL/min). An empty pan was used as reference. X-ray diffraction (XRD) was performed using a diffractometer (D8-Advance, Bruker, Ettlingen, Germany) equipped with an Apex II CCD detector. The crystallinity state of CsA in raw CsA powder and CsA-NCs was analyzed. The X-ray source was Kα radiation from a copper target with a graphite monochromator at a wavelength of 1.54 Å. Standard runs using a 40 kV voltage, a 40-mA current, and a scanning rate of 2°/min over a 2θ range of 5–45° was performed.

### 2.5. Particle Size Stability Studies

This study aimed to investigate the effects of particle size on cellular uptake and transport across Caco-2 cell monolayer of CsA-NCs. So, it is necessary to investigate the stability of particle size in experimental process. The particle size of CsA-NCs was studied at 4 °C and room temperature for 3 months. The particle size of CsA-NCs was also investigated in RPMI 1640 medium (containing 10% FBS) and HBSS. The mean particle size and PDI of all samples were analyzed in triplicate and reported as the standard deviation.

### 2.6. Dissolution Studies

HBSS containing 0.01% SDS was employed as the dissolution medium to evaluate the dissolution profiles of CsA-NCs with different particle sizes. Briefly, CsA-NCs (containing 20 μg CsA) were dropped into 900 mL dissolution medium. The temperature was maintained at 37 ± 0.5 °C and stirred at 100 rpm. Samples (5 mL) were collected and replaced with the equal volume of fresh medium at predetermined time intervals 5, 10, 20, 30, 40 and 50 min). The samples were filtered through a membrane filter of 0.1 µm pore size and the filtrate was assayed for CsA content by validated HPLC method.

### 2.7. HPLC Analysis

An Agilent 1200 HPLC system equipped with a DAD detector was used to assay the content of CsA. The absorbance wavelength was set at 214 nm. The mobile phase was a mixture of phosphate acid (pH 2.5) solution and acetonitrile at a 10:90 *v/v*. A CAPCELL PAK C18 column (5 µm, 4.6 mm × 250 mm, Shiseido, Japan) was used with a flow rate of 1 mL/min and the column temperature was maintained at 70 °C using a column heater.

### 2.8. In Vitro Cellular Uptake and Monolayer Permeation

#### 2.8.1. Cell Culture

The Caco-2 cells were cultured by following the regular procedures in 1640 medium at 37 °C, 90% RH and 5% CO_2_ in T-25 flasks. The culture medium was changed every other day. The cells were passaged every 4–6 days after dissociation with 0.25% trypsin/0.02% EDTA solution when the cell fusion rate reached 85%.

#### 2.8.2. In Vitro Cytotoxicity

Caco-2 cells were seeded in 96-well plates at a density of 5 × 10^4^ cells in 200 μL medium per well and incubated for 48 h. The culture medium was removed, then culture medium containing various concentrations (from 100 to 800 µg/mL) of CsA-NCs was added to the cells. After incubation for different times (from 2 to 8 h), the medium was replaced with fresh medium containing 1 mg/mL of MTT, and the cells were further incubated for 4 h. Then the supernatant was removed, and the MTT formazan crystals were dissolved in 100 µL DMSO under gentle shaking for 30 min at room temperature. The absorbance was measured using a Spectrophotometer (3020, Thermo Fisher Scientific Oy, South San Francisco, CA, USA) at 562 nm. Cell viability was calculated by measuring the absorbance.

#### 2.8.3. Cellular Uptake and Uptake Mechanism Analysis

Caco-2 cells were seeded in 24-well plates at a density of 1 × 10^6^ cells per well and incubated for 48 h to allow the cells to attach to the wells. The cells were treated with 200 μL HBSS alone for 30 min. Then, the cells were treated with 200 μL HBSS containing various concentrations (from 100 to 300 µg/mL) for 3 h to study the effect of concentration on the cellular uptake. The cells were treated with 200 μL HBSS containing 200 µg/mL CsA-NCs for different time intervals (0.5, 1.0, 2.0, 3.0 and 4.0 h) to investigate the effect of time on the cellular uptake. At certain time point, the HBSS was removed, and the cells were rinsed quintic with PBS, lysed with PMSF and RIPA buffer (PMSF: RIPA = 1:100). 50 μL cell lysate was mixed with 200 μL acetonitrile. The mixture was vortexed and centrifugedto obtain the supernatant for LC-MS/MS analysis.

These results were expressed as the amount (µg) of drug per mg of total cellular protein.

To evaluate the specific mechanisms involved in the uptake mechanism of the CsA-NCs, Caco-2 cells were incubated with CsA-NCs (200 μg/mL) at 4 °C for 2 h. Besides, the cells were preincubated with HBSS containing different endocytosis inhibitors for 30 min, including 30 μM chlorpromazine, 30 μM nystatin, 10 mM methyl β-cyclodextrin, 2.5 mM amiloride, 10 μM Cytochalasin D, 25 μm monensin sodium, and 50 μM chloroquine at 37 °C, respectively. Controls were prepared without the inhibitor pre-incubation at 37 °C. Then, CsA-NCs (200 μg/mL) were added into this cells at 37 °C and incubation for 2 h. The culture medium was removed, and the cells were washed with PBS for 5 times, lysed with PMSF and RIPA buffer (PMSF: RIPA = 1:100). 50 μL cell lysate was mixed with 200 μL acetonitrile. The mixture was vortexed and centrifuged. Then, the supernatant was obtained for LC-MS/MS analysis. Results were expressed as relative uptake percentage compared to the control.

Determination of CsA concentration in the cell lysing reagent by LC/MS/MS.

#### 2.8.4. Transport of CsA-NCs across the Caco-2 Cell Monolayers

Caco-2 monolayers were used to evaluate the ability of transmembrane transportion for CsA-NCs. Briefly, Caco-2 cells were seeded onto the apical (AP) side of Millicell-CM cell culture plates (Millipore Corp., Bedford, MA, USA) in a density of 1 × 10^5^ cells/cm^2^, and cultured for 21 d under 5% CO_2_, 90% relative humidity, and 37 °C. The apical (AP) and basolateral (BL) compartments contained 0.2 and 1.3 mL of culture medium, respectively. The culture medium was replaced every other day for the first week and daily thereafter. The trans-epithelial electrical resistance (TEER) was measured, and a threshold value of 500 Ω/cm^2^ was set for transmembrane studies. The culture medium was replaced with warm HBSS (37 °C). The cell monolayer was equilibrated at 37 °C for 30 min before conducting the transport studies. HBSS was removed and 200 µL HBSS containing CsA-NCs (40 µg/mL CsA) was added to the AP compartments, while 1.3 mL HBSS was filled into the BL side. At predetermined time points (0.5, 1.0, 1.5 and 2 h), aliquots (100 µL) were withdrawn from the BL side, and an equivalent volume of HBSS was added to maintain a constant volume. The CsA concentration in the samples was determined by LC-MS/MS. The apparent permeability coefficient (*P_app_*, cm/s). *P_app_* was calculated using the following Equation [24]:

$$P_{app}=\frac{dQ}{dt}\times \frac{1}{{AC}_{0}}$$

where *dQ*/*dt* is the transport rate (µg/min), *C*_0_ is the initial drug concentration on the apical side (µg/mL), and *A* is the surface area of the membrane filter (0.3 cm^−2^).

### 2.9. LC-MS/MS Measurement

CsA concentration in the samples was measured using an Agilent 6460A (Palo Alto, CA, USA) triple quadrupole LC-MS/MS system with an Agilent 1200 series combined LC system. The Agilent source parameters were a capillary voltage of 4500 V, gas temperature was 180 °C, gas flow was 13 L/min, nebulizer gas pressure was 18 psi. The mass transitions were m/z 1219.7 → 1202.8 for CsA (fragmentor voltage 180 V, collision energy 30 eV), m/z 1233.8 → 1216.8 for CsD (fragmentor voltage 180 V, collision energy 30 eV). The autosampler temperature was set at 10 °C and the column oven temperature was set at 60 °C. Analyses were performed with a 3 μm 100 × 2.1 mm Thermo HyPURITY C18 analytical column (Waltham, MA, USA). The mobile phase consists of 90% methanol and 10% 10 mM ammonium formate buffer (pH 3.5) with a flow of 0.3 mL/min and a run time of 3.0 min.

## 3. Results and Discussion

### 3.1. Characterizations of the CsA-NCs

CsA-NCs with different particle sizes prepared by the wet bead milling method. The mean particle size, PDI and zeta potential were shown in Table 1. The results demonstrated that the average particle size of CsA-NC (450 nm), CsA-NC (240 nm), and CsA-NC (165 nm) were 492.5 ± 3.2 nm, 246.1 ± 1.2 nm and 165.9 ± 4.6 nm, respectively. In addition, the PDI and the negative zeta potential of the CsA-NCs were been found below 0.2 and approximately −20 mV, respectively. It’s reported that a suitable size distribution and zeta potential could minimize Ostwald maturation and improve the stability of the nanocrystals [25].

**Figure 1:**
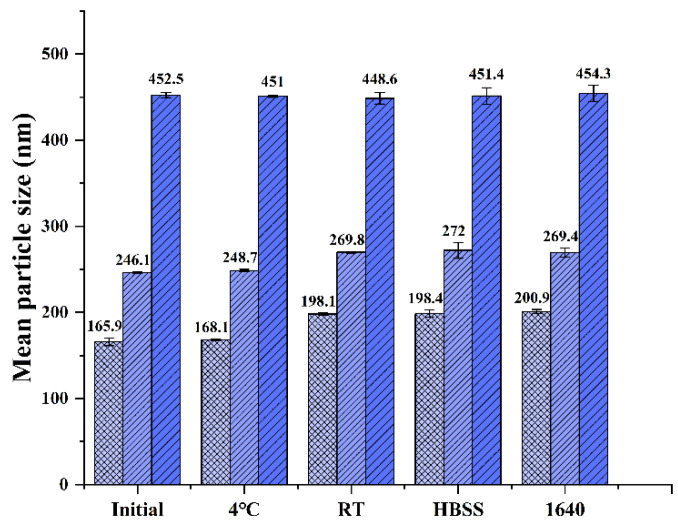
Particle size stability of CsA-NCs in different condition. Initial: 0 day after preparation; 4 °C: stored at 4 °C for 3 months; RT: stored at room temperature for 3 months; HBSS: mixed with HBSS at 37 °C for 12 h; 1640: mixed with 1640 medium at 37 °C for 12 h (*n* = 3, means ± SD).

It is important that the nanocrystals remain stable in experimental process. As showed in Figure 1, no significant changes in particle size of the CsA-NCs were observed within three months at 4 °C and room temperature (RT), indicating that the particle size of CsA-NCs was sufficiently stable in storage. Particle size of the CsA-NCs did not change significantly after the CsA-NCs mixed with RPMI 1640 medium (containing 10% FBS) and HBSS for 12 h at 37 °C, which suggests that they could remain stable when incubated with cells.

**Figure 2:**
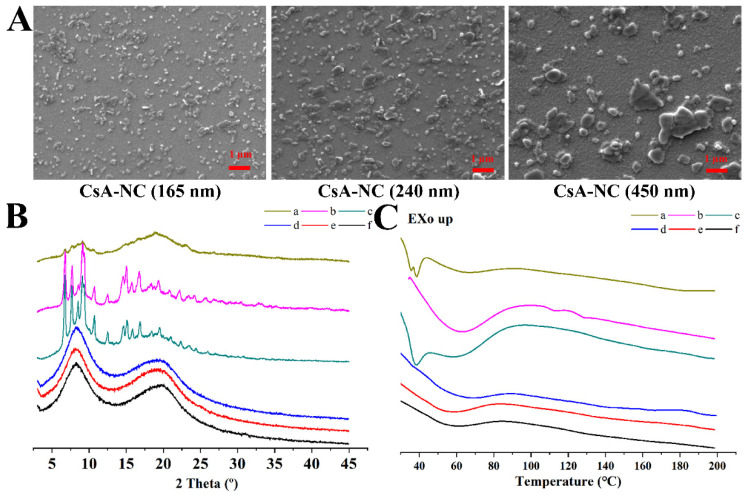
Physicochemical characterizations of various formulations. (**A**) SEM of various CsA-NCs; (**B**) XRD spectra; (**C**) DSC thermograms. (a) stabilizers; (b) free CsA; (c) physical mixture; (d) CsA-NC (450 nm); (e) CsA-NC (240 nm); (f) CsA-NC (165 nm).

SEM photographs (Figure 2A) showed that the morphologies of CsA-NCs displayed a near spheroid shape, and the smaller the particle was, the more uniform it was distributed. In the process of wet bead milling method, strong shear force and attrition force of the yttrium-stabilized zirconium oxide grinding beads in the wet media mill significantly impacted upon the macroparticles and broken them into multiple microparticles.

XRD was used to detect crystallinity of drug in the samples (Figure 2B). Obvious peaks were observed in X-ray diffractograms of the free powder of CsA and the physical mixture. These peaks revealed that there was a crystalline structure in the free CsA and the physical mixture. In CsA-NCs, XRD data showed no crystallization peaks of CsA, which demonstrated amorphous nature of the drug in the nanocrystals. The changes in crystallinity of CsA were considered relevant to the milling process [26,27].

As shown in Figure 2C, the results of the DSC scanning showed the melting temperature of each sample during the heating process. For raw CsA, one strong endothermic peak and two weak endothermic peaks were observed at 80 °C and approximately 107–132 °C [28], respectively. The strong endothermic peak was attributed to be an enthalpic relaxation and the two weak endothermic peaks were attributed to be the solid-to-liquid transition [23]. The two weak endothermic peaks were not observed for physical mixture, which may be due to some influence from stabilizers. For the physical mixture and stabilizers, endothermic events were observed at approximately 37 °C that were associated with the intrinsic melting point of TPGS (37–41 °C) [29]. This peak is absent in the CsA-NCs, it can be concluded that TPGS was absorbed by the surface of nanoparticles in molecular form. The enthalpic relaxation endotherm for the CsA-NCs became weak, which was related to the presence of water.

### 3.2. Dissolution Studies

**Figure 3:**
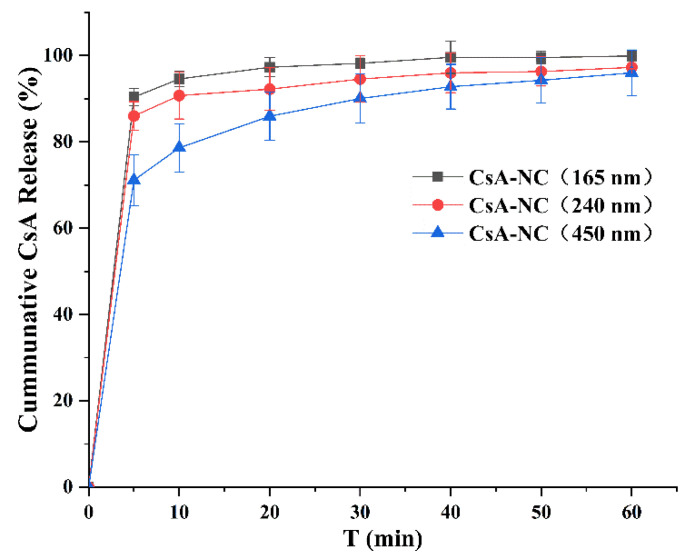
Dissolution profiles of CsA-NCs in HBSS containing 0.01% SDS. (*n* = 3, means ± SD).

Figure 3 showed the dissolution profiles of CsA-NCs with different particle sizes in HBSS containing 0.01% SDS over the time period of 50 min. 165 nm CsA-NC showed highest dissolution rate (94.6% in the first 10 min), while the release percentage of CsA from 240 nm and 450 nm CsA-NCs were approximately 90.7% and 78.6% within 10 min, respectively. At other time intervals, the dissolution rate of CsA-NC (165 nm) was also greater than that of CsA-NC (240 nm) and CsA-NC (450 nm). Faster dissolution rate of CsA in nanocrystals with smaller particle size might be attributed to the increase in surface area and the decrease diffusion layer thickness [30].

### 3.3. In Vitro Cellular Uptake and Uptake Mechanism Analysis

#### 3.3.1. Cytotoxicity

**Figure 4:**
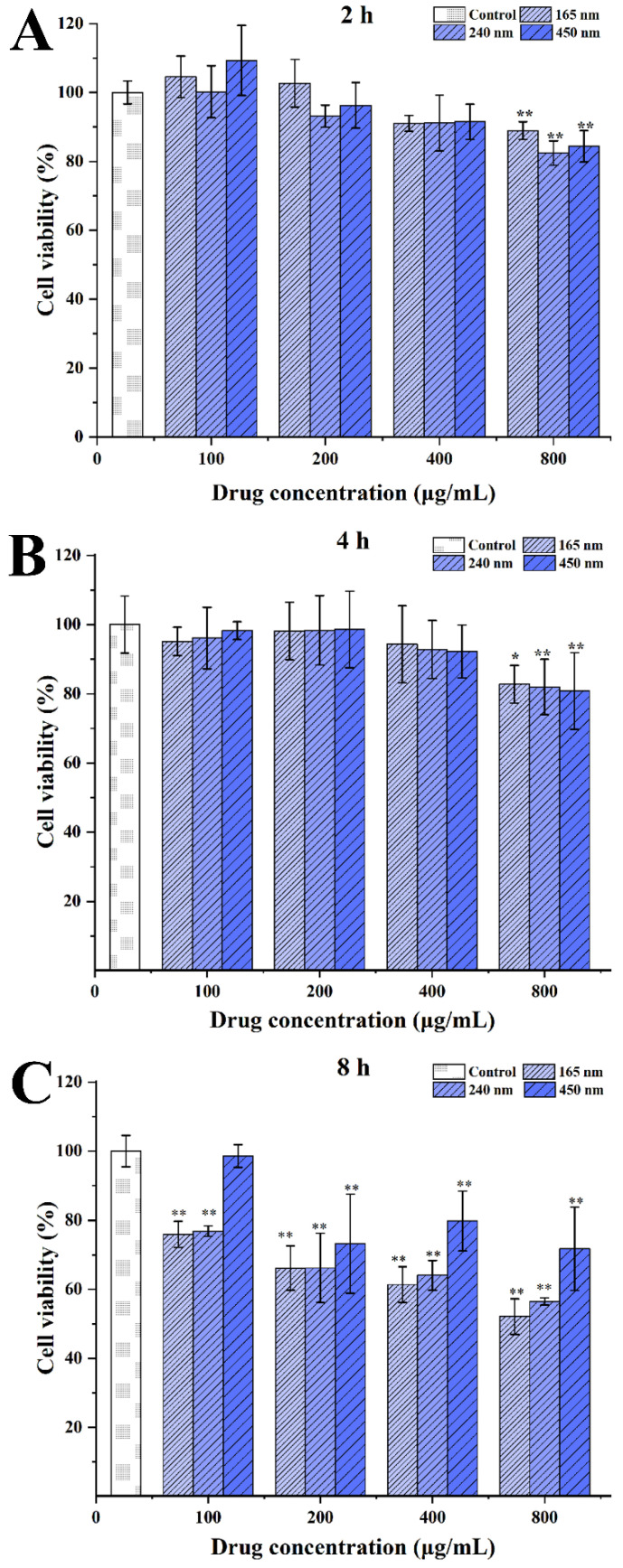
Concentration-dependent cytotoxicity of different sized CsA-NCs in Caco-2 cells at 2 h (**A**), 4 h (**B**) and 8 h (**C**) (*n* = 6, means ± SD). * represents significantly different compared with control (*p* < 0.05); ** represents extremely significantly different with control (*p* < 0.01).

The cytotoxicity of the CsA-NCs to Caco-2 cells was determined by MTT assays to determine the optimum incubation dose and time in the following experiments. As shown in Figure 4, all of the tested formulations inhibited Caco-2 cells replication in a concentration- and time-dependent manner. In this experiment, the cytotoxicity of CsA solution (1640 culture medium containing 10% DMSO) was also determined and found obvious cytotoxicity to Caco-2 cells at the same concentration (Data was not showed). This is because CsA has a low solubility, 10% DMSO was added in 1640 culture medium before CsA was formulated as a solution, which resulted in obvious toxicities to Caco-2 cells [31]. The CsA-NCs significantly inhibited the viability of Caco-2 cells at concentration of 800 µg/mL or for the incubation time of 8 h. Thus, a concentration of <800 µg/mL of CsA-NCs and a time < 8 h were used in the following experiments.

#### 3.3.2. Effect of Particle Size, Incubation Concentration and Time on Uptake of CsA-NCs by Caco-2 Cells

**Figure 5:**
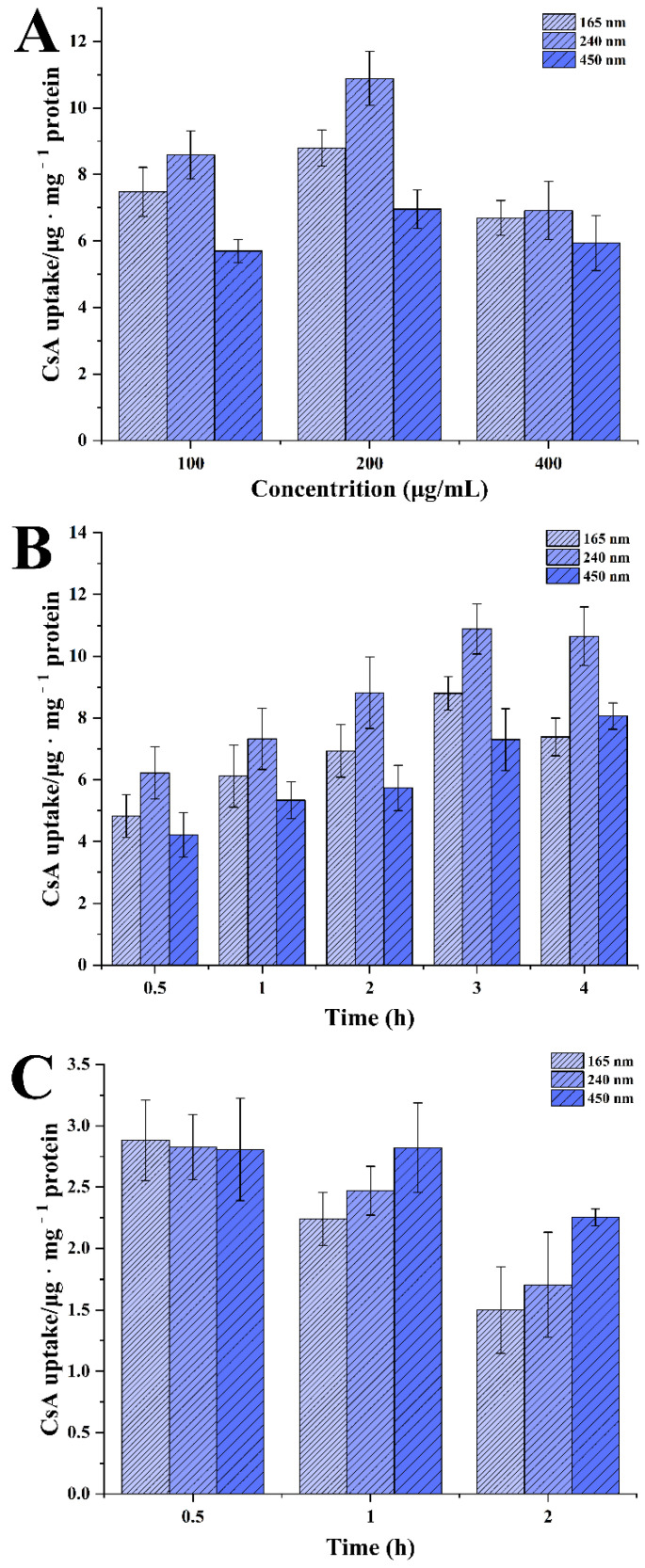
Uptake of CsA-NCs in Caco-2 cells after the incubation of different concentration (**A**) and time (**B**), and additional 2 h incubation with only fresh medium after 3 h of treatment (**C**). (Mean ± SD, *n* = 6 for each group).

To calculate the uptake of CsA-NCs in Caco-2 cells, the amount of CsA internalized by Caco-2 cells after co-culture with CsA-NCs at different concentration or time was quantitatively measured by LC-MS/MS. As shown in Figure 5A, the uptake for 3 h exposure of CsA-NCs at below 400 μg/mL increased with concentration. And no further increase was observed for the uptake of CsA-NCs for the concentration of 400 μg/mL. For 200 μg/mL, the uptake of CsA-NCs increased to 8.8 ± 0.5, 10.9 ± 0.8 and 6.9 ± 0.6 μg · mg^−1^ protein for 165 nm, 240 nm and 450 nm CsA-NCs, respectively. Interestingly, for different concentrations uptake experiments, 450 nm and 240 nm CsA-NCs showed the lowest and highest uptake in Caco-2 cells, respectively. Figure 5B showed that the uptake of CsA-NCs by the Caco-2 cells increased with the incubation time within a 3 h period at 200 μg/mL. The uptake of CsA-NCs had no further increase beyond 3 h incubation period. And at various points in time, the cellular uptake of CsA-NCs in Caco-2 cells 450 nm and 240 nm CsA-NCs also showed the lowest and highest uptake in Caco-2 cells, respectively.

The saturation of cell uptake of CsA-NCs was observed with the increase of incubation concentration and time. This plateau effect is in agreement with some previous studies [10,32,33], suggesting the involvement of active transport as the uptake process [34]. It has been proposed that the size of the particles plays a key role in their adhesion to and interaction with the biological cells [35]. Figure 5A and B showed that it was CsA-NC of 240 nm but not CsA-NC of 165 nm showed the highest uptake in Caco-2 cells. These results conflict with other reported research [36], in which the cell uptake of microparticle is dependent upon the particle size, the smaller particles are, the greater uptake they have [37]. It was reported that if the nanoparticles are too small, their surface energy would not be enough for the needed bending energy in the endocytosis process. Moreover, smaller CsA-NCs would result into faster drug dissolution rate and CsA would be secreted from the Caco-2 cells by P-glycoprotein [38], as a classical transported substrate of p-glycoprotein. These results suggested there might be an optimal particle size rang for Caco-2 cellular uptake [32,39,40,41].

Although the endocytosis of CsA-NCs was mainly emphasized, the exocytosis should also be considered in this process. The uptake greatly decreased for all three sizes after an additional incubation time of 0.5 or 2 h in only HBSS alone, compared with the uptake at 3 h (Figure 5C). After another 2 h incubation, the uptake of CsA-NCs in Caco-2 cells decreased to 1.5 ± 0.35, 1.7 ± 0.43 and 2.2 ± 0.07 μg mg^−1^ protein for 165 nm, 240 nm and 450 nm, respectively. And the content of CsA in Caco-2 cells showed the faster decline with smaller particle size of CsA-NCs. The uptake of CsA-NCs in the cells illustrate drastically decreased, indicating the dissolution (and possible exocytosis) of the nanocrystals [6,7], and different size possibly displayed different exocytosis efficiency [40].

#### 3.3.3. Possible Uptake Pathways for Different-Sized CsA-NCs by Caco-2 Cells

**Figure 6:**
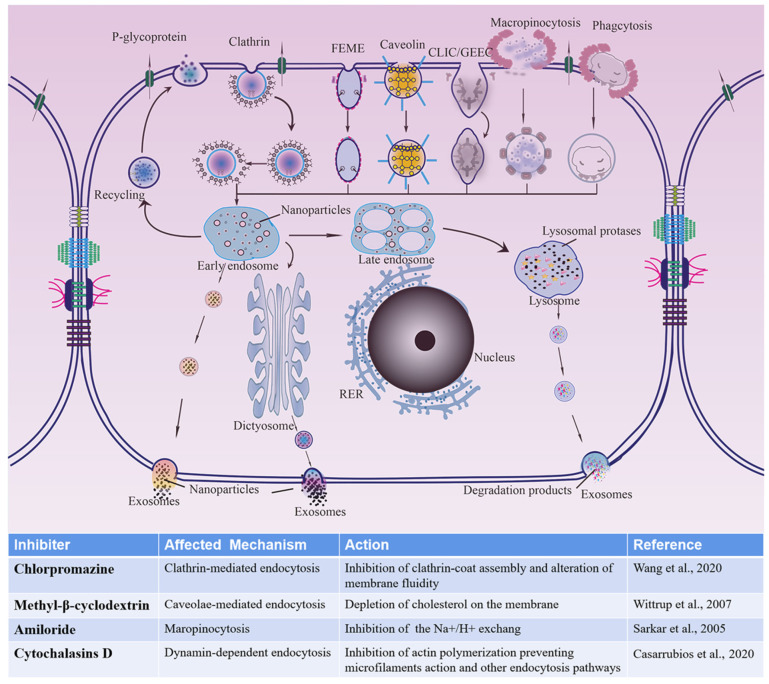
Schematic diagram about cellular uptakes of CsA-NCs into Caco-2 cells and inhibitory effects of several endocytosis inhibitors. FEME: clathrin-independent/dynamin-dependent endocytosis; CLIC/GEEC: clathrin-independent/dynamin-independent endocytosis; RER: rough endoplasmic reticulum. Wang et al., 2020 [25], Wittrup et al., 2007 [48], Sarkar et al., 2005 [49], Casarrubios et al., 2020 [50].

The cellular internalization pathways for different-sized CsA-NCs were studied by using different endocytic inhibitors. Energy dependent uptake of CsA-NCs experiments were performed at 4 °C. The effect of inhibitors on cells activity was examined at predetermined concentration and no significant differences were found compared with the control group (data not shown). As showed in Figure 6, possible cellular uptake mechanisms of particles and inhibitory effects of inhibitors were introduced in detail. The results showed the uptake of the three sized CsA-NCs was significantly inhibited by every of these inhibitors, which indicated that the overall uptake of CsA-NCs in Caco-2 cells was a combination of active mechanisms including clathrin, caveolin/lipid raft, actin dependent pathways and micropinocytosis [42].

**Figure 7:**
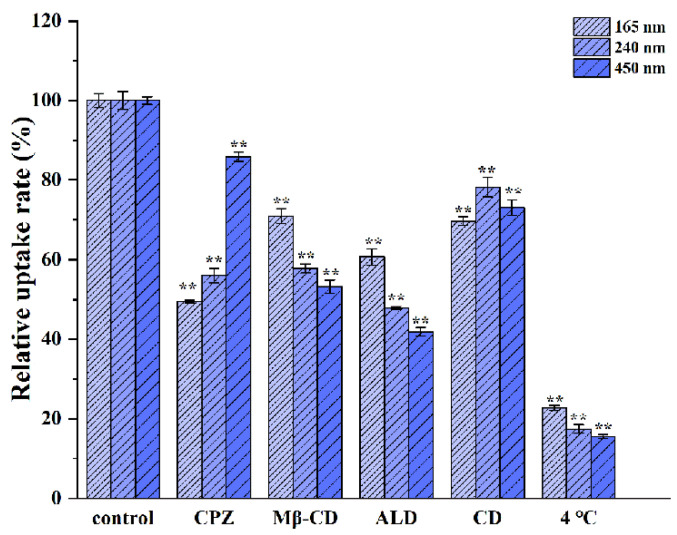
The relative uptake rate of different-sized CsA-NCs after inhibitor treatments in Caco-2 cells. Cells were separately pretreated with CPZ, Mβ-CD, ALD and CD for 30 min before the addition of CsA-NCs at 37 °C for 2 h or only treated with CsA-NCs at 4 °C for 2 h (Mean ± SD, *n* = 3). Abbreviations: CPZ, chlorpromazine hydrochloride; Mβ-CD, methyl-β-cyclodextrin; ALD, amiloride; CD, cytochalasins D. ** represents extremely significantly different with control (*p* < 0.01).

As shown in Figure 7, after incubation with chlorpromazine, CsA-NC (165 nm) and CsA-NC (240 nm) uptake were reduced to 49.49 ± 0.3% and 56.06 ± 1.78%, respectively, both of which were lower than that of CsA-NC (450 nm) of 85.82 ± 1.23%, indicating that CsA-NC (165 nm) and CsA-NC (240 nm) were more associated with clathrin-mediated internalization. Those particles with a diameter of 200 nm or less were mostly processed along the pathway of clathrin mediated endocytosis, consistent with previous results [8,25,43]. The internalization of all three-sized CsA-NCs was obviously inhibited by methyl-β-cyclodextrin. Furthermore, the inhibition of methyl-β-cyclodextrin on CsA-NC (165 nm) uptake was weakest, and its uptake was increased by 1.33-fold compared with that of CsA-NC (450 nm). It has been well-established that some of different types of endocytosis are susceptible to cholesterol depletion [8,43,44]. Uptake of particles with 200–500 nm mainly depended upon caveolae mediated endocytosis [25]. Cellular uptake of particles of 250 nm to 3–5 μm can be achieved through the nonspecific uptake of extracellular fluid- micropinocytosis [45]. Macropinocytosis can been inhibited by amiloride interdicting the amiloride-sensitive Na+/H+ exchanger in the plasma membrane [46]. Actin polymerization is also involved in regulation of micropinocytosis which can be inhibited by actin polymerization inhibitors (cytochalasin D) [47]. After pretreatment with amiloride, the uptake of CsA-NC (450 nm) and CsA-NC (240 nm) were 41.88 ± 1.03% and 47.84 ± 0.37%, respectively, both of which was lower than that of CsA-NC (165 nm) of 60.65 ± 2.04%. Actin dependent pathways were involved in the internalization of three-sized CsA-NCs as observed by the cytochalasin D inhibition (69.7 ± 1.13% uptake for the 165 nm, 78.1 ± 2.44% uptake for the 240 nm and 73.1 ± 1.95% uptake for the 450 nm CsA-NCs). The inhibitory effect of amiloride on cell uptake of CsA-NCs was stronger than cytochalasin D, which indicated that macropinocytosis is more dependent upon Na+/H+ exchanger compared with actin polymerization. The absorption of CsA-NC (450 nm), CsA-NC (240 nm) and CsA-NC (165 nm) by Caco-2 cells at 4 °C decreased to 22.74 ± 0.64%, 17.44 ± 1.05% and 15.53 ± 0.46%, respectively, indicating an energy-dependent process of internalization.

### 3.4. Transport of CsA-NCs across the Caco-2 Cell Monolayers

**Figure 8:**
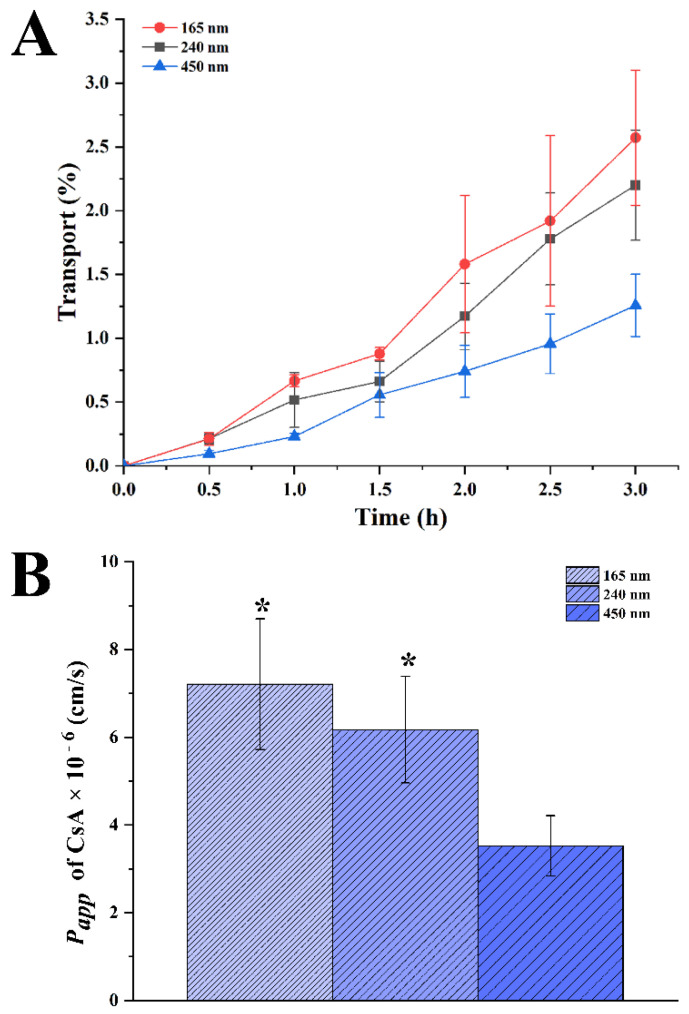
(**A**) Relative cumulative transport of CsA in CsA-NCs across Caco-2 monolayer; (**B**) Apparent permeability coefficient *(P_app_*) of CsA across a Caco-2 cell monolayer (*n* = 3, Mean ± SD). * represents significantly different compared with CsA-NC (450 nm) (*p* < 0.05).

The capacity of CsA-NCs to permeate an intestinal epithelial barrier was determined with Caco-2 cell monolayers. There was no significant difference in TEER value of cell monolayers before and after incubating with CsA-NCs, indicating that cell monolayers were intact during the experiment [51]. But CsA solution (HBSS containing 2% DMSO) significantly reduce the TEER value of cell monolayer (Data was not showed), which could be caused by the DMSO and high concentration of CsA molecules. Therefore, CsA solution was not used as a control in this experiment. As shown in Figure 8A, the transport of CsA-NCs in the basolateral side (BL) increased with the extension of time, and the smaller the particle size, the better the transmembrane transport. The amount of CsA in the BL side of monolayers treated with CsA-NC (165 nm) was highest among the three-sized of CsA-NCs at each time point. After incubating for 3 h, the transport of CsA in the BL side was 2.57 ± 0.53%, 2.20 ± 0.43% and 1.26 ± 0.24% for CsA-NC (165 nm), CsA-NC (240 nm) and CsA-NC (450 nm) respectively. The *P_app_* of CsA from the apical side to the basolateral side was calculated after incubating Caco-2 cells with CsA-NCs for 0 to 3.0 h. As shown in Figure 8B, the *P_app_* of the CsA-NC of 165 nm [(7.21 ± 1.49) × 10^−6^ cm/s] was higher than the corresponding value of CsA-NC of 240 nm [(6.17 ± 1.21) × 10^−6^ cm/s], and significantly higher than the corresponding value of CsA-NC of 2967 nm [(3.53 ± 0.68) × 10^−6^ cm/s].

Decreasing particle size would be responsible for improving transport of CsA-NCs through monolayer by improving dissolution rate and saturation solubility. In addition, Compared with large size nanoparticles, small size nanoparticles are more easily transported across monolayers [52]. Though the efflux of p-glycoprotein, diffusion rate was the dominant factor for transmembrane transport of CyA-NCs at higher concentrations, the p-glycoprotein was saturated [38]. Therefore, CsA-NCs with small particle size have stronger transmembrane transport capacity than those with large particle size.

## 4. Conclusions

The results taken together shown that the dissolution rate, uptake and transmembrane transport of CsA-NCs are correlated with particle sizes. Our results also revealed that there might be an optimal particle size rang for Caco-2 cellular uptake of CsA-NCs. Multiple endocytic pathways may be involved in CSA-NCs by inhibitory assay. Although there are multiple uptake pathways for all three-sized particles, different inhibitors have different inhibition rates on uptake of CsA-NCs with different particle sizes. Our data indicated that the decrease of particle size benefited improving the dissolution rate in vitro and transport of CsA through Caco-2 cell monolayer. Our study provided very important information to understand interaction mechanism between CsA-NCs and Caco-2 cells.

## Figures and Tables

**Table 1:** Particle size, PDI and zeta potential of CsA-NCs (*n* = 3, mean ± SD).

CsA-NCs	Mean Particle Sizes (nm)	Polydispersity Index (%)	Zeta Potential (mV)
450 nm	452.5 ± 3.2	0.19 ± 0.016	−22.3 ± 1.2
240 nm	246.1 ± 1.2	0.15 ± 0.014	−21.3 ± 0.4
165 nm	165.9 ± 4.6	0.13 ± 0. 023	−24.3 ± 0.4

## Data Availability

Not applicable.
